# Applying Parse's Theory in Caring for a Depressed Patient Following Hysterectomy With Oophorectomy: A Case Study

**DOI:** 10.1002/cnr2.70301

**Published:** 2025-08-05

**Authors:** Arian Mobasheri, Azar Darvishpour

**Affiliations:** ^1^ Healthy Ageing Research Centre Neyshabur University of Medical Sciences Neyshabur Iran; ^2^ Noncommunicable Diseases Research Center Neyshabur University of Medical Sciences Neyshabur Iran; ^3^ Social Determinants of Health Research Center, Trauma Institute Guilan University of Medical Sciences Rasht Iran; ^4^ Department of Nursing, Zeyinab (P.B.U.H) School of Nursing and Midwifery Guilan University of Medical Sciences Rasht Iran

**Keywords:** case study, depression, home care, hysterectomy, nursing, oophorectomy

## Abstract

**Background:**

Depression is a common complication in women undergoing hysterectomy. By utilizing nursing theories in patient care, improved care standards, more affordable care costs, and greater quality of life can be achieved. Parse's theory of human becoming is among the nursing theories prioritizing each individual's conception of quality of life as a crucial nursing goal. This study aimed to apply Parse's theory of human becoming to the care of a depressed patient following a hysterectomy with oophorectomy.

**Case:**

The participant was a 44‐year‐old woman who underwent hysterectomy with bilateral oophorectomy on January 1, 2023, following a diagnosis of grade 1 right ovarian cancer. After three days of hospitalization, she returned home to live with her husband and four children. Postoperative care was provided by a home care nurse—also the researcher—who observed signs of emotional distress and depressive symptoms, including social withdrawal, irritability, and loss of interest in daily activities. The nurse implemented nursing interventions grounded in Parse's Human Becoming Theory, including therapeutic communication, mindfulness practices, journaling, and involving the family in emotional support. Data were analyzed using Parse's qualitative research methodology (2011).

**Conclusion:**

Based on the first principle of Parse's theory (meaning), the findings showed that patients' meaning and values of life changed from “disappointment” to “hope.” In the second principle (rhythmic patterns), contradictory patterns of “dependency‐nondependency” and “hope‐disappointment” were identified. Based on the third principle (transcendence), with the actual presence of a nurse and the implementation of care and treatment, the patient's mental state switched from “depression and despair” to “hope and happiness.” This research found that Parse's theory of nursing was effective in alleviating depression in patients following hysterectomy with oophorectomy surgery. This study also highlights the importance of utilizing nursing theories in patient care.

## 1 Introduction

Hysterectomy, a surgical procedure involving the removal of the uterus with or without the cervix, and with or without the ovaries, is one of the most common surgeries performed on nonpregnant women, ranking as the second most common surgery after cesarean section [1]. Malignant conditions, including endometrial carcinoma, malignant ovarian tumors, cervical cancer, and malignancies of adjacent organs, are primary indications for hysterectomy [2]. However, this surgery has a profound impact on women's sexual characteristics, as the uterus is often symbolic of femininity, fertility, and childbirth, and is closely tied to a woman's sense of identity and self‐concept. Consequently, the loss of the uterus can be perceived as a loss of femininity, representing a significant change in a woman's physiological and reproductive functions, as well as her sense of youth, energy, and activity [1, 3]. It is therefore not unexpected that some studies have demonstrated that hysterectomy can be a potent trigger for stress and psychological problems in women [1]. Furthermore, the removal of one or both ovaries via oophorectomy has also been reported to increase the risk of depression [4]. Consistent with this, previous longitudinal studies have shown that hysterectomy with oophorectomy is associated with an increased risk of depressive symptoms in women postsurgery [3, 5].

The primary goal of treatment is to facilitate individuals in achieving their fullest potential of independence, functioning, and self‐acceptance [6]. Following a hysterectomy, women often experience a compelling desire for support from their family members, healthcare providers, and colleagues [7]. To effectively address this issue, nurses are required to be aware of these potentially distressing situations. Consequently, nursing interventions should be implemented to address these challenges. A thorough analysis of the psychosocial needs of patients undergoing hysterectomy is also essential for nurses. Nurses should strive to provide patients with sufficient information and deliver suitable emotional and social support. Furthermore, nurses can conduct assessments to identify and treat specific psychological issues in high‐risk groups, and they can also offer nursing interventions or make necessary referrals [8]. Home health care, as a method of community‐based care, is a vital component of healthcare delivery [9]. Home health care plays a crucial role in the delivery of healthcare services, occurring in the patient's living environment with the participation of the patient and their family members, and has many benefits for the family and the patient [10].

The recent history of the nursing profession has demonstrated that nursing care theories and models can be utilized to provide comprehensive care [11]. Indeed, more advanced care standards, affordable costs, and greater quality of life can be achieved through the application of nursing theories in patient care [12]. Nursing theories are considered vital for supporting professional independence, goal coherence, and professional communication, and provide a basis for practice [13]. Parse's theory of human becoming is a nursing theory that considers each person's conception of quality of life as one of the most important nursing goals [14]. According to this theory, a person is a single being with free will, exchanging with the environment [15]. This theory views individuals as self‐sufficient beings who choose the path of their destiny among the existing conflicting patterns [16]. Based on this theory, nurses, through their presence, help patients make the best decisions about their health [17]. Parse first published her theory under the title “Man‐Living‐Health” in 1981, and in 1992, she changed the theory to “the theory of human becoming” [18]. This theory highlights the importance of health in human life, emphasizing the individual's role in shaping their destiny [19]. According to Parse's theory, humans coexist with the rhythmic patterns of the world [13]. Individuals are open beings who freely choose the meanings of situations and accept responsibility for decisions [18]. There are nine fundamental assumptions: four about the human and five about becoming. Additionally, three assumptions about human beings were synthesized from these nine assumptions [19]. According to Parse, individuals are always changing and evolving. Nurses help patients have the right attitude toward themselves and their relationships by acknowledging and addressing their fears, worries, and ways of building their hopes. Nurses do not show the way to patients because Parse believes that the patient knows the way and that the nurse helps them discover the right path [15].

The first principle of Parse's theory (1981) implies that humans choose personal meaning for life experiences. Embodiment, valuation, and manifestation are concepts that give structure to this principle. The second principle of this theory describes the contradictory patterns of communication with the environment, including self‐disclosure–concealment, enablement–restriction, and communication–separation. These patterns coexist simultaneously and are not mutually exclusive. The third principle concerns transformation and implies that humans are always changing as they strive to achieve their hopes and dreams. The concepts included in this principle include the exercise of power, creation, transformation, or change [20].

These principles demonstrate the concept of paradox as a basis of human life. Paradoxes are not problems to be solved, but rather natural rhythms of life. Furthermore, they are a way of giving meaning to human life [21].

Based on phenomenological ideology, human transformation theory posits that humans live through purposeful choices in unique circumstances to emphasize what is personally important to them [20]. Using Parse's insight, living with a serious illness can be considered a unique experience. As people choose personal meaning for their experiences, they engage in rhythmic contradictions that propel them forward and create new opportunities for transformation [22].

Parse's theory has been used as a guide for nurses worldwide, and many studies have been conducted based on this theory, most of which were conducted abroad [17, 23, 24]. For example, Alanizi used Parse's theory to explain the concept of “shameness” [25]. Doucet used Parse's theory to care for families with a member with dementia [26]. Hart used a case study to show how to use Parse's theory for long‐term care [27]. Doe et al. conceptualized Parse's theory of human becoming to create a novel idea about hope [28].

Despite numerous studies, a review of the literature indicates that limited studies have been conducted in the field of applying this theory. A review of the literature also reveals that this theory has not been used in patients suffering from depression after hysterectomy with oophorectomy. Therefore, the purpose of this study was to apply Parse's theory of human becoming in the care of a patient suffering from this disease.

## 2 Methods

### 2.1 Study Design

This study was conducted as a case study in 2023, utilizing Parse's model of human becoming to care for a patient suffering from depression following a hysterectomy with oophorectomy. A case study is a research method that focuses on the in‐depth examination of a case, subject, or phenomenon [29], enabling a deep and multifaceted exploration of complex issues in real‐life events [30].

### 2.2 Introducing the Case

The participant was a 44‐year‐old woman who underwent a hysterectomy with oophorectomy on January 1, 2023, due to right ovarian cancer. She was discharged after 3 days of hospitalization and returned to her home, where she lived with her husband and children. The patient had four children (two girls and two boys).

The patient's medical history revealed that she was diagnosed with a malignant tumor on December 23, 2022, based on tests and ultrasound results. Her physician determined that chemotherapy was not necessary after the operation, as the tumor was grade 1. The patient's postoperative care was managed by a home care nurse, who visited her at home to change dressings, administer medications, and provide postoperative care. The home care nurse, who is also the researcher, has a general nursing degree and is currently pursuing a master's degree in nursing. She has received education and training in mental health nursing as part of her general nursing program, which enabled her to provide supportive care to the patient and recognize signs of depression.

During these visits, the researcher observed noticeable mental changes in the patient. The patient sometimes exhibited nervousness and moods of depression. Her family members also reported similar changes, stating that she had become withdrawn, lost interest in activities she previously enjoyed, and had difficulty communicating with others.

To assess the patient's depression status, the researcher administered the Beck Depression Inventory (BDI) questionnaire. The BDI is a 21‐item questionnaire developed by Beck et al. that measures the severity of depressive symptoms. Each item is scored on a scale of 0–3, with higher scores indicating more severe symptoms [31]. The patient's score of 18 indicated mild depression.

The BDI has been widely used and validated in various populations, including American adults and Iranian samples, with reported reliability coefficients of 0.90 and 0.86, respectively [32, 33]. The validity of the questionnaire was also acceptable in this research.

### 2.3 Applying Parse's Theory

To care for the patient and facilitate her recovery, the researchers applied Parse's theory. To identify the patient's needs and problems, a semistructured interview was conducted. This type of interview is suitable for qualitative research due to its flexibility and depth [34]. The interview commenced with the open‐ended question, “What changed in your life after the operation?”

To conduct the research based on the principles of Parse's theory, questions were asked about the purpose and meaning of life, the ability of compatibility, and the power of change, which were used during the care sessions. The primary questions based on the principles of Parse's theory were as follows:

The First Principle of Parse's Theory: The Purpose and Meaning of Life.

How would you describe your current health state?

What are your beliefs and values regarding this disease?

What does life mean to you now?

What helps improve meaning in your life?

What do you feel has changed in your life?

How has your relationship with others changed?

What is the reason for this change?

What is the most important thing for you right now?

The Second Principle of Parse's Theory: The Ability of Compatibility.

How did you think before this experience?

What were you thinking about during this time of solitude?

What needs to happen to improve your situation?

What did you use to do when you were in a bad mood?

The Third Principle of Parse's Theory: The Power of Change and Attitude to the Future.

What is your current concern?

What do you have in mind to change your life?

What actions have you taken regarding these thoughts?

What is your attitude toward the future?

What are your hopes and dreams?

Nine sessions were held with the patient and her family, with six sessions dedicated to the patient and three sessions dedicated to her family. The initial interview with the patient was conducted on March 3, 2023, at her home through a semistructured face‐to‐face interview, lasting 65 min.

A plan was developed to implement Parse's theory based on the interviews conducted in the first session. According to the first principle of Parse's theory, individuals' views or perceptions of the world create realities, which are co‐created with others. According to Parse's second principle, individuals create patterns in their daily lives, which concern meanings, concepts, and personal values. Attempts were made to lead the subsequent sessions according to the patient's records and interests.

The nursing interventions in this study included therapeutic communication with the patient to strengthen positive thinking, increase hope, and change the meaning of life. During the interview, the researchers discovered that the patient used to read psychological books before her illness and held an international meditation certificate. Thus, the researchers focused on the patient's abilities and asked her to read daily and perform meditation exercises. The patient was also encouraged to record past positive events as a task in a notebook. The patient wrote about her abilities and the positive events of the past, mentioning her pleasant experiences in the next meeting.

At every session, the nurse reviewed the patient's assignments, discussed them with her, and provided guidance. Additionally, during these meetings, the patient discussed other problems and concerns about herself and her illness with the nurse.

Three meetings were also held with the patient's family regarding necessary training on treating the patient. They were taught to assign tasks to her that she was able to accomplish, increasing the patient's self‐confidence. According to the patient's condition, tasks were assigned in a way that did not cause her fatigue.

### 2.4 Data Analysis

In this study, data analysis was conducted using the qualitative analysis‐synthesis process of Parse's research methodology [35]. This approach aims to uncover the underlying structure of individuals' or groups' lived experiences, thereby enhancing our understanding of the human experience [36]. The methodology comprises four distinct steps. First, participants who are willing to describe the phenomenon under investigation through various means, such as words, drawings, metaphors, stories, or other media, are selected. The subsequent step involves interaction through dialogue, which entails the researcher's presence and engagement with the participant, characterized by undivided attention. The third step encompasses extraction and synthesis, a process that involves in‐depth contemplation, describing participants, and translating abstract concepts into scientific propositions. This is achieved by transcribing participants' recorded descriptions, extracting and synthesizing core concepts, compiling statements that capture the essence of each participant's experience, combining main concepts from the formulated statements, and ultimately constructing the structural composition of the lived experience of the core concepts. The final step involves heuristic interpretation, which expands nursing knowledge through structural integration and conceptual interpretation, informed by theories of human evolution [37]. Table 1 provides a summary of the primary codes, subcategories, and main category that emerged from this study.

**TABLE 1:** Summary of the primary codes and subcategories of the main category “disappointment and change in understanding the meaning and values of life” based on Parse's theory.

Quotations	Primary codes	Subcategories	Main category
I feel that I have lost my femininity, and I can no longer be as useful as before.	The feeling of loss of femininity	Conflict in the mental image	Disappointment and change in understanding the meaning and values of life
I am not a woman anymore, it bothers me.			
The scar on my stomach is very ugly.	Disorder in Mental image		
I do not like to communicate with family and friends anymore.	Lack of interest in communicating with files and friends	Withdrawal from others	
I do not like others to look at me with pity.	Not interested in the pitiful look of others on her		
I did not hurt anyone why this happened to me.	Thinking about the cause of the disease	The sense of karma of the disease^a^	
This world is no longer worth it	Devaluing life	Feeling depressed	
I do not feel like doing things.	Boredom		
I do not like any clothes.	Losing interest in daily issues		
Skin creams have become unimportant to me.			
I will get osteoporosis in the long run with the decrease in my female hormones.	Making negative changes in physical condition in the future	A negative view of the future	
My skin will age.			
The future is meaningless to me.	The meaninglessness of the future		
I am afraid that I will get severe depression like my mother.			
I would like to visit my doctor soon for a check‐up.	Fear of recurrence of the disease in the future		
What should I do if my disease recurs?			
My stomach starts to hurt when I do the slightest thing.	Physical inability to do work as before	A feeling of disturbance in the state of physical health	
I should not have sex for 6 months.			
I wish I could have another child.			
I do not like my husband to see my stomach.	Shame about losing beauty in her husband's eyes		
I am more upset for my husband than for myself.			

^a^ Karma means that everything we do comes back to us; In such a way that if we do a good deed, good will be returned to us and if we do a bad deed, bad will be returned to us.

### 2.5 Rigor

To ensure the scientific accuracy and reliability of this research, we employed a multifaceted approach to data collection, incorporating interviews, observations, and note‐taking. Additionally, we utilized theoretical synthesis, soliciting the expertise of two specialists in data interpretation, as well as incorporating their suggestions and solutions. Furthermore, we allocated sufficient time to conduct interviews, verify findings with participants, and meticulously document each stage of the research, including data collection, analysis, and interpretation. This rigorous approach ensured the accuracy and reliability of our findings.

## 3 Results

This study was grounded in the three principles of Parse's theory. The first principle, which focuses on the search for meaning in life from the patient's perspective, was explored. According to this theory, “disappointment and change in understanding the meaning and values of life” were observed to be prevalent. Following in‐depth interviews with the patient and observation of her searching for meaning in life, the researchers found that she had experienced disappointment and a shift in her understanding of the meaning and values of life. Specifically, the loss of her uterus and ovaries led her to feel that she had lost her femininity and was no longer as useful as before, resulting in feelings of worthlessness and a negative outlook on the future.

The patient's interview revealed the following sentiments:

*I no longer feel like a woman, as I have lost my uterus and ovaries. What can I do about this large scar on my stomach? I am ashamed in front of everyone, especially my husband. This is unfair to him. If I can cope with all of this, I will never be able to cope with my inability. No matter what I do, I experience stomach pain. My two aunts died of cancer, and I fear that my illness will return, leaving my children without a mother. I rushed through my life, and maybe there was a better way. I read online that this procedure would cause me to age quickly, and my skin would become wrinkled due to the decrease in hormones. I am devastated. Oh God, what a disaster. I wish I could turn back time. I wish I had taken better care of myself. These ‘what ifs’ are destroying me. I feel like God has turned his face away from me and is no longer paying attention to me*.

The second principle of Parse's theory, which explores the contradictory and rhythmic patterns in the patient's disease process, was also examined. The disjunctive rhythm describes how an individual moves simultaneously with some phenomena while moving away from others. The patient's interview revealed that she was not interested in communicating with friends and family, yet she had a strong sense of dependence on them. She was also more inclined toward spirituality and avoided material possessions. She made an effort to take better care of herself. The following statements from her interview illustrate this:

*I don't want my friends to see me in this situation, especially now that I've read online that I'm aging prematurely due to the decrease in hormones. However, my children were very supportive during this time, especially my daughter, who was by my side day and night, which means that while I was distant from my friends, I had a strange attachment to my family*.

*I used to enjoy hosting big parties and having luxurious furniture, but these things are no longer important to me. I now appreciate the simple things in life. I used to avoid the sun, but now it's a symbol of hope for me, and I sit under it for hours. I used to do everything to please others, but now I understand that one's life is fragile, and I am more important than anyone or anything*.

The patient also exhibited contradictory and mixed feelings of hope and despair, as evident in the following statements:

*Sometimes I think it was good that I underwent surgery and had the tumor removed, while other times I wonder if I was too hasty in my decision, and perhaps if I had consulted another doctor, I might not have needed the surgery and would not have lost my uterus and ovaries, she said. These conflicting emotions are also troubling me*.

In accordance with the third principle of Parse's theory, interventions were implemented based on the previous two principles, which led to changes in the patient's mental state, transforming her from a state of depression and despair to one of happiness and exaltation of human personality. These interventions varied depending on the patient's condition, and different methods were employed to facilitate transformation. According to Parse's theory, the researchers designed interventions tailored to the patient's interests and personality, aiming to alter her perspective and improve her health status. Following the nursing interventions, the patient's perspective on her disease underwent a significant shift. In an interview, she stated:

*I am grateful that I made a timely decision about my illness; God was with me, and I was fortunate to avoid the fate of my aunts, who were diagnosed at a late stage. Now, I am relieved that I do not require chemotherapy, and I can be with my husband and children*.

*After meditating, I experienced a sense of light emerging from the darkness of my soul. I can attest that this positive thinking and meditation have been instrumental in my recovery, and I feel more relaxed. Additionally, the support of my family has been invaluable in my return to a normal life. I feel that I have regained my former self and even surpassed my previous state; I no longer avoid social interactions, and everything is enjoyable for me*.

Meetings were also conducted with the patient's family members, which led to a change in their behavior and significantly contributed to her recovery process. Following the training sessions with the family and a subsequent interview with the patient's husband, he reported:

*I used to think that providing for my wife's material needs was sufficient, but now I understand that she requires my emotional support and love. I must admit that after our conversation (with the nurse) and following your advice, I feel that I love her more than before. I am sincerely grateful*.

The children were also interviewed, and each of them expressed satisfaction with the care measures. For instance, the patient's son mentioned:

*I told my mother that I craved a particular meal, and if possible, could she prepare it for me. While she was cooking, we were by her side, providing her with everything she needed, so she wouldn't have to exert herself. After the meal was ready, I praised her extensively, and she seemed very happy*.

## 4 Discussion

This case study, grounded in Parse's theory of human becoming, explored the care of a patient experiencing depression following a hysterectomy with oophorectomy. The patient's disappointment and altered understanding of life values were transformed through the application of Parse's theory, which ultimately shifted her attitude toward life.

According to Parse, humans possess the capacity for self‐directed choice, continually reviewing and re‐evaluating their situations to imbue their lives with meaning. The structure of meaning, as posited by Parse, is comprised of three inherent concepts: visualization, valuation, and language learning [16]. The findings of this study, guided by the *first principle of Parse's theory*, revealed that the participant's experience of depression following oophorohysterectomy was characterized by a loss of femininity, diminished self‐confidence, reduced ability to engage in previously enjoyed activities, and impaired communication with others. The participant's values and perception of life were also negatively impacted, leading to feelings of disappointment. However, the application of Parse's theory facilitated a shift in the participant's attitude toward life. Consistent with this finding, Jouybari et al. [15] demonstrated the effectiveness of Parse's theory in enhancing quality of life and improving health outcomes in patients with burn injuries. Similarly, Hold and Blake's study, grounded in the first principle of Parse's theory, utilized phototherapy to improve the life expectancy of a patient, highlighting the significance of patient interest in guiding therapeutic interventions [38]. In fact, in this research, the nurse has a meaningful discussion with the patient, asking the patient questions and obtaining information that can improve the patient's quality of life; additionally, the researcher refrains from offering advice, instead empowering the patient to discover solutions by herself. By asking questions, the nurse helps the patient discover the solution by herself. In the present study, the patient was first interviewed, and a history of her interests and abilities was taken. Therefore, it was determined what the patient should do to improve her condition. In fact, in this research, based on Parse's theory, care for patient recovery was personalized.

According to the *second principle of Parse's theory*, individuals exhibit a paradoxical pattern of dependence and independence, hope, and disappointment. The nurse's diligent efforts to identify and address these conflicting patterns facilitated a deeper understanding of the patient's experiences. Furthermore, the patient's expression of these issues provided a sense of emotional relief. The patient's ambivalence toward her cancer treatment, feeling both relieved and saddened by the operation, exemplified the enabling‐limiting rhythm. While eschewing materialistic pursuits, she found solace in spiritual connections. Conversely, despite the physical distance from relatives and friends, she remained emotionally dependent on her family, illustrating the connection‐separation rhythm of Parse [20]. Hold and Blake's study on a patient with HIV applied Parse's second principle, highlighting the significance of nurse presence. The patient's behaviors reflected patterns of disclosure‐concealment, enablement‐restriction, and communication‐separation, as he balanced individuality with conformity in his relationships and interactions [38]. Alanizi further illuminated the concept of shame through the connection‐separation rhythm, where individuals may feel disconnected from the present moment, even in the presence of others [25].

In accordance with the *third principle of Parse's theory*, which emphasizes the creation of unique approaches in the transformative process, interventions were designed and implemented based on the patient's interests and abilities. A novel approach to healing was developed through the incorporation of family support and educational resources on psychology and meditation. This novel approach facilitated the patient's transformation and empowerment. Consistent with this finding, Melnechenko's study demonstrated that the application of Parse's theory enabled cancer patients to reframe their concept of death, replacing it with a sense of hope [20]. According to Hold and Blake's study, the third principle of Parse's theory, which posits power as a transformative force, was evident in the patient's experience of making difficult decisions. The patient's performance and patience declined, prompting him to seek change. Although he initially hoped to return to his preoperative state, he eventually accepted that his life had been irreversibly altered and adapted to the new circumstances. Through the exercise of power and creation, the patient's life was transformed, as he leveraged his abilities and interests to overcome depression and increase personal peace and communication [38].

The present study found that patient interest can be a valuable tool in the treatment of depression. Similarly, a study by Razzaghian and Bigdeli demonstrated that systematic motivational counseling based on patients' abilities and interests can be an effective strategy for preventing depression in individuals with cancer and may even be more effective than pharmacological interventions alone [39]. Furthermore, Doucet's research on a family with severe dementia, which utilized the human becoming family model, highlighted the significant impact of family presence on enhancing quality of life [26]. The findings of this study demonstrate the potential of Parse's theory to improve depression in patients who underwent hysterectomy with oophorectomy. The results highlight the importance of tailoring care to the individual needs and circumstances of each patient and the role of the nurse in facilitating this process. It should be kept in mind that care should be tailored to each individual's condition, and it is not possible to write a single prescription or nondrug treatment for all depressed patients. Additionally, in this study, it was found that the nurse not only serves as a guide but also helps the patient find the meaning of the situation and synchronizes the rhythmic patterns of transformation in the individual. By applying Parse's theory, nurses can help patients find meaning and purpose in their experiences and promote the transformative processes that underlie health and well‐being. We hope that this study will contribute to the development of more effective and personalized interventions for depression in patients who underwent hysterectomy with oophorectomy.

One of the notable strengths of this study is its utilization of nursing theories in facilitating the recovery of a patient with depression. This approach is particularly significant, given the dearth of attention often afforded to nursing theories in clinical practice and treatment protocols. The findings of this study highlight the importance of incorporating nursing theories into care and treatment, and it is hoped that they will stimulate increased interest and attention to the application of these theories in future research and practice. By using a theoretical framework to guide care, nurses can provide more holistic and patient‐centered interventions that address the unique needs of each individual. Future research should build on these findings and explore the application of Parse's theory in other populations and settings, with the ultimate goal of improving patient outcomes and advancing the science of nursing practice.

The main limitation of this research is that this study presented the results of an intervention on depression in a cancer patient who underwent a hysterectomy with oophorectomy, and the findings may not be generalizable to all patients with depression. Therefore, conducting research on more samples is recommended.

## 5 Conclusion

This study demonstrates the benefits of using Parse's theory to alleviate depression in patients who have undergone hysterectomy with oophorectomy. The findings emphasize the significance of nursing theories in guiding care and promoting positive patient outcomes. We hope that this study will inspire further research and encourage nurses to incorporate theoretical frameworks into their practice, ultimately leading to improved care and better health outcomes for patients.

## Author Contributions

A.M. was the first researcher involved in the study design and the data collection, analysis, and interpretation. A.D. is the corresponding author. She supervised the data collection and analyzed the data. All the authors have read and approved the manuscript.

## Ethics Statement

The study was performed under the ethical standards established in the Declaration of Helsinki and its later amendments or comparable ethical standards. After selecting the participant, the researcher explained the objectives of the study to the patient, and written and verbal consent informed was obtained from the patient. The patient was assured that her information would remain confidential and that she had the right to withdraw from the study at any time during the study.

## Consent

Written and verbal informed consent were obtained from the patient prior to the publication of her deidentified case report.

## Conflicts of Interest

The authors declare no conflicts of interest.

## Data Availability

The data that support the findings of this study are available on request from the corresponding author. The data are not publicly available due to privacy or ethical restrictions.
